# High Level of HBV DNA Virus in the Breast Milk Seems not to Contraindicate Breastfeeding

**DOI:** 10.4084/MJHID.2015.042

**Published:** 2015-07-01

**Authors:** A Montoya-Ferrer, AM Zorrilla, J Viljoen, JP Molès, ML Newell, P Van de Perre, E Tuaillon

**Affiliations:** 1 Université Montpellier 1, Inserm U1058, UM1 UFR Pharmacie, 15 avenue Charles Flahault, 34090, Montpellier, France; 2 Département des Maladies Infectieuses et Tropicales, UMI 233, Centre Hospitalier Régional Universitaire de Montpellier, 80 av Augustin Fliche - 34295 Montpellier, France; 3 University of Florida College of Medicine, 1600 SW Archer Rd, Gainesville, Florida 32603, United States; 4 Africa Centre for Health and Population Studies, University of KwaZulu-Natal, R618 en route to Hlabisa, Somkhele, A2074 Rd, Mtubatuba 3935, Durban, South Africa; 5 Faculty of Medicine, University of Southampton, Mailpoint 887, Institute of Developmental Sciences Building, Southampton General Hospital, Tremona Road, Southampton, Hants SO16 6YD, United Kingdom; 6 Département de Bactériologie-Virologie, Centre Hospitalier Universitaire de Montpellier, 191 Avenue Doyen Giraud, Montpellier, 34295, France

## Dear Editor

Breastfeeding is recommended by the World Health Organization regardless of mother’s hepatitis B virus (HBV) status since breast milk is not considered as an efficient pathway for HBV perinatal transmission.^1^ However, concerns about a possible risk of HBV transmission through breastfeeding arises under conditions favoring an increase of HBV DNA viral load in breast milk, particularly among unvaccinated children.^2^,^3^

We present here a case of an HIV-HBV co-infected mother who exposed her child to a significant HBV infectious inoculum via breast milk, likely due to a hepatitis flare or an acute HBV infection.

The mother-child couple was enrolled in the Kesho Bora study; a randomized controlled trial evaluating maternal prophylactic strategies to reduce HIV transmission through breastfeeding.^4^ At week 6 post-delivery, a > ten fold elevation of alanine transaminase (ALT: 750 IU/L) was observed in a 20 years old mother. This mother was included in the control arm receiving short course antenatal zidovudine and single-dose intrapartum nevirapine in accordance with per national guidelines at the time. Serological testing confirmed HBV infection (positivity for HBsAg) and ruled out hepatitis A and C infections. Serum and breast milk samples were retrospectively assessed for for HBV DNA levels at week 2 at week 2 and 12 using a standardized qPCR method (HBV Generic PCR, Omunis, Montpellier, France). Significant levels of HBV DNA were found in both left and right breast milk samples at week 2 post-delivery (> 10^3^ log_10_ HBV DNA IU/mL) whereas, ALT level was normal (12 IU/ml). HBsAg levels were also quantified in breast milk (>10^1^ IU/ml) using a quantitative assay (Architect HBsAg QT assay, Abbott, Chicago, IL). A dramatic decrease in the levels of HBV DNA and HBsAg was observed in both serum and breast milk at week 12 post-delivery (Table 1). Serum and breast milk became negative for HBsAg 6 months after delivery without detection of anti-HBs antibody. The newborn was infected neither by HIV nor by HBV despite a vaccine schedule starting at week 6. Protective anti-HBs titers were observed in response to HBV vaccination (>ten mIU/mL) at month 12. We estimate that the intestinal mucosa of the infant was in contact with more than one million HBV particles/per day based on a daily consumption of 300 ml during the first weeks of life. Although HBV infection is a major health concern worldwide, very few studies have explored HBV DNA in breast milk.

HBV DNA has been detected in the colostrum of mothers tested positive for HBe antigen using PCR and Southern blot hybridization^5^ and in banked human milk samples using a nested PCR^6^.

Recently, HBV DNA was tested in serum and breast milk using a standardized quantitative PCR method in HIV-HBV coinfected mothers who were under lamivudine-containing antiretroviral regimen from the 25th week of pregnancy^7^. At delivery, 11/26 (42%) women had significant HBV DNA levels in serum (ranging from 3.3 to >8 log_10_ IU/mL) that steadily decreased at months 1, 3, and 6 postpartum (median of 5.2, 4.5 and 2.8 log_10_ IU/mL respectively). HBV DNA was detected in breast milk from three HBV viremic women (33%) and four out of 24 breast milk samples (17%). When detectable, HBV DNA levels in breast milk were low in all cases ranging from 1.18 to 2.20 log_10_ IU/ml, suggesting that level of HBV DNA in breast milk might always be negligible. The rate of HBV infection was also assessed among 23 HIV-HBV exposed children along the two years study period. Diagnosis of transient HBV infection was made in four children whereas other three children acquired HBV infection during the postnatal period (first HBsAg positive test at 12 months in one case and 24 months in the other two cases). Pirillo et al observed that those 7 children were exposed to the highest HBV infectious inoculum, not only at delivery, but also during all the postnatal period, since their mothers presented the highest levels of HBV DNA in serum. However, no correlation was found between HBsAg positivity and HBV DNA levels in breast milk.

In our case, exposure to a larger amount of HBV infectious particles via breast milk was detected, but the child, who was correctly vaccinated, was not infected with HBV. This result seems to confirm that breastfeeding is not an effective route of HBV transmission during the perinatal period, as previously reported in clinical studies.^2^,^3^ However, in contrast with the results of Pirillo et al, our data show that HBV exposition via breast milk could be significant, especially during a high HBV replicative phase. HBV exacerbations or hepatitis flares often occur among chronic HBV infected mothers after pregnancy leading to higher levels of HBV replication in the blood^8^,^9^. In our case, an accurate differential diagnosis between an acute HBV infection and a hepatitis flare could not be made since the time of HBV infection was unknown. A typical elevation of ALT 6 to 10 weeks after delivery preceding the upsurge of HBV DNA levels suggested a hepatitis flare.^10^ However, this mother also experienced HBsAg clearance within six months, as often occurs in acute HBV infections.^11^

Hepatitis flares or acute HBV infections in chronic HBV infected patients do not usually required antiviral treatment since most of the episodes resolve spontaneously.^12^ In pregnant women, the risk of fulminant or severe hepatitis following hepatitis flares or acute HBV infections does not appear increased (9). However, as in general population, those episodes may progress to a fulminant liver failure and must be treated with nucleot(s)ide analogs and be evaluated for liver transplantation.^12^,^13^ Due to its safety and better resistance profile, Tenofovir is currently the antiviral of choice for the treatment of severe acute HBV infection, and its efficacy has been demonstrated even in cases in which previous nucleoside analog treatment as lamivudine had failed.^14^ Hence, Tenofovir provides a better efficiency during a hepatitis flare or a post-delivery acute HBV infection.

Exposure to a significant HBV infectious inoculum through breastfeeding should not always be considered negligible in children from HIV-HBV coinfected mothers who do not receive any active antiviral treatment against HBV. However, cumulative exposure during lactation does not seem to contraindicate breastfeeding among correctly vaccinated children despite the immaturity of their intestinal mucosa.

## Figures and Tables

**Table 1:** 

	W2 post delivery			W12 post delivery		
	Plasma	BM right	BM left	Plasma	BM right	BM left
**HBV DNA PCR (IU/mL)**	7.36 × 10^9^	6.60 ×10^3^	4.27 × 10^3^	3.69 × 10^2^	<1 × 10^2^	<1 × 10^2^
**HBsAg detection**	Positive	Positive	Positive	Positive	Negative	Negative
**HBsAg level (IU/mL)**	1.25 × 10^5^	1.10 × 10^1^	1.51 × 10^1^	2.32 × 10^1^	<0.05	<0.05
**HBeAg detection**	Positive	Positive	Positive	Positive	Negative	Negative
**HIV RNA (copies/mL)**	1.82 × 10^4^	2.81 × 10^2^	4.71 × 10^3^	2.25 × 10^4^	1.24 × 10^3^	6.67 × 10^2^
**ALT (IU/L)**	26			17		
**CD4 T cells count (cells/μL)**	426			311		

BM: acellular breast milk

<1 × 10^2^ HBV DNA IU/mL: detected but not quantified
